# Bloody Ventriculography: Intracerebral Hemorrhage Artistically Casting the Ventricular System’s Anatomy Into a Bird’s Head

**DOI:** 10.7759/cureus.23165

**Published:** 2022-03-14

**Authors:** Afaf Shaabi

**Affiliations:** 1 Neurological Surgery, King Fahad General Hospital, Jeddah, SAU

**Keywords:** basal ganglia hemorrhage, hypertension, computed tomography, radiographic anatomy, hydrocephalus, intraventricular hematoma

## Abstract

Causes of intracerebral hemorrhage are mostly attributed to vascular anomalies or hypertension. It causes significant morbidity and mortality worldwide. This illustrative case report presents an interesting computed tomography image of hyperacute basal ganglia hemorrhage with intraventricular extension artistically casting the ventricular system. The intraventricular hemorrhage was moderate upon presentation; the blood had delineated the anatomic details of the ventricular system. It closely resembles a Crax rubra’s head in the sagittal view. We believe that this radiographic finding is not seen frequently, especially early in the course of the condition. However, it could serve as an intriguing and reproducible sign of the radiographic anatomy of the ventricular system.

## Introduction

Causes of intracerebral hemorrhage are mostly attributed to vascular anomalies, cerebral amyloid angiopathy, or hypertension [1]. It causes significant morbidity and mortality worldwide with a high fatality rate [2]. Risk factors amongst affected populations are mostly lifestyle-related and modifiable [1]. Most intraventricular hemorrhage (IVH) results from a dissecting intraparenchymal hematoma [3] such as in this case. Hydrocephalus is a well-known complication of IVH with complex pathophysiology involving cerebrospinal fluid (CSF) reabsorption hindrance by the extravasated blood [4,5]. This condition can be fatal if not recognized early to allow timely neurosurgical diversion of CSF [6]. To date, the paucity of options and clear consensus for the surgical management of asymptomatic hydrocephalus in IVH cases is a real challenge [6,7]. In the emergency setting, non-contrast computed tomography (CT) of the brain remains the imaging modality of choice to detect intracranial hemorrhage [8]. This illustrative case report is a “grateful reminder” of the earliest attempts of the brilliant neurosurgeon Dr. Walter Dandy in 1918, and Leonardo da Vinci before Dr.Dandy to mold the ventricular anatomy using contrast media, air, and wax [9,10]. In this case, the blood outlined the detailed anatomy of the CSF-filled cisterns of the ventricular system rendering this brain CT image an invaluable teaching tool. It also resembles a crested bird’s head most strikingly a Crax rubra. The author suggests the name “Crax rubra sign” for this finding. To the best of the author's knowledge, there is no case in literature with this unique, ominous depiction of ICH-IVH surviving fatal clinical outcome. This inevitably queries the independent significance of cast forth ventricle as a predictor of clinical outcome in IVH [11].

## Case presentation

**Figure 1:**
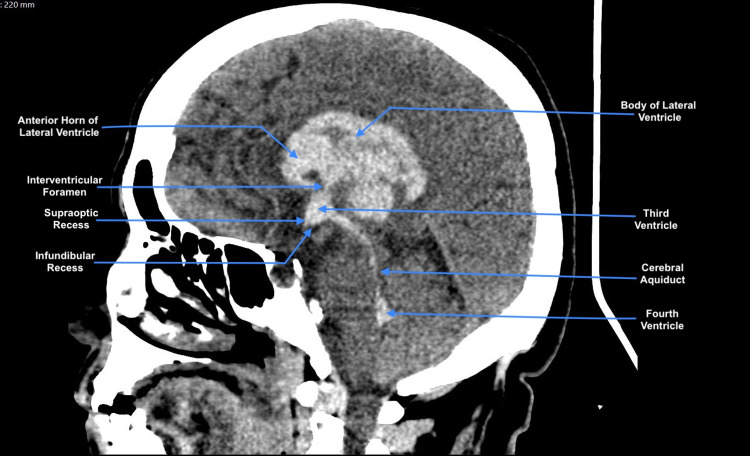
Non-contrast CT image of the brain, sagittal view. The labeled hyperdense area is a right-sided intraparenchymal hematoma casting the ventricular system and outlining the details of its anatomy [12].

A 66-year-old woman presented to the emergency department with a complaint of dizziness along with left arm numbness and weakness. The initial blood pressure in the emergency department was 150/82 mmHg. Other vital signs were as follows: heart rate, 109 beats per minute, regular; temperature, 36.8°C; respiratory rate, 18 breaths/minute; initial Glasgow coma scale (GCS) was 15/15, with equal round pupils of 3 mm and reactive to light. Her left arm was hypotonic with muscle strength of 0/5 and diminished sensation. No other neurological deficits were noted. Her past medical history was remarkable for uncontrolled hypertension, morbid obesity, and previous ischemic stroke six months ago. She denied using anticoagulant or antiplatelet medications. Non-contrast CT brain image reported large right-sided intracerebral hematoma within the basal ganglia (Figure 1). Her GCS deteriorated to 7/15 gradually during the subsequent weeks mainly due to COVID-19 pneumonia. Apart from the resolving intracerebral hematoma, there was no neurosurgical explanation for the GCS decline.

## Discussion

The initial CT image of the brain (Figure 1) showed large right-sided intracerebral hematoma epicentered within the basal ganglia, measuring 3.8 × 3.1 × 4.3 cm in maximum anteroposterior, transverse, and craniocaudal dimensions. The heterogenic density denoting the hyperacute nature of the bleed, correlates with poor clinical outcomes [13]. It was associated with extension to the intraventricular cavities (frontal and occipital horns as well as the body of the right lateral ventricle, body, occipital horn of the left lateral ventricles, and third and fourth ventricles). There was significant mass effect related to the hematoma and perihematomal white matter edema causing 0.5 cm midline shift and compressing the ipsilateral cortical sulci in axial cuts (not shown here). The radiodensity of the hematoma in the CT image has proven to be an important parameter in predicting clinical outcomes [14]. Moreover, this interesting CT image highlights the significance of each anatomical configuration of the ventricular system in different individuals. Which in infants, for example, served as an invaluable tool to predict the development of hydrocephalus after intraventricular hemorrhage (IVH) [15,16].

**Figure 2:**
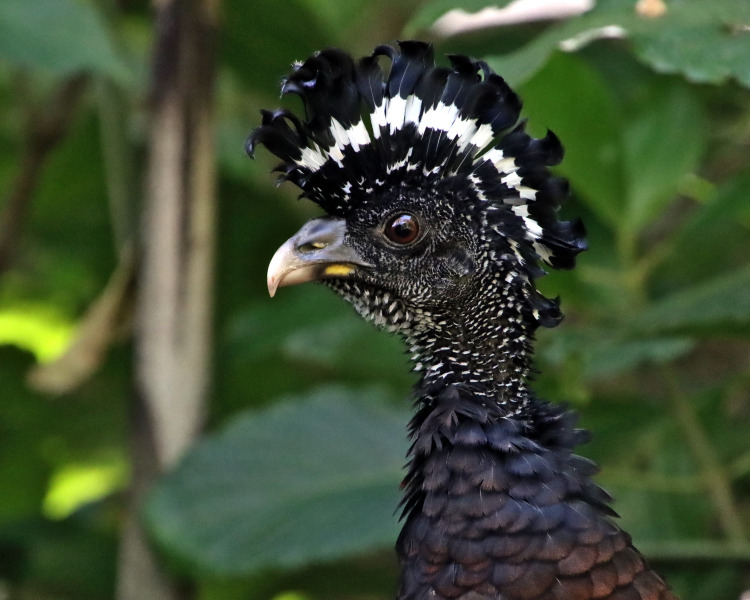
Crax rubra. A photograph by Zinn, N. (2020). Great Curassow (Crax rubra) 2048×1638 [17] with permission.

**Figure 3:**
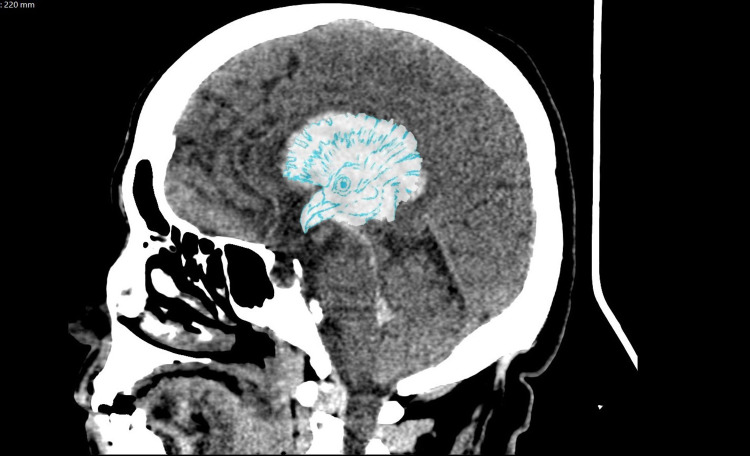
Crax rubra sign. Illustrated non-contrast CT brain image sagittal view of a right-sided intraparenchymal hematoma matching the transparent sketch of Crax rubra bird. The head crest fans out the lateral ventricle, the eye is precisely situated in the interthalamic adhesion, and the bird’s peak spans the third ventricle from every angle. The word “rubra” means red in Latin which conveniently describes the blood-filled cerebrospinal fluid (CSF) spaces in intraventricular hemorrhage (IVH).

The patient was managed conservatively with medical blood pressure control and follow-up CT imaging of the brain in the intensive care unit. Cerebral CT angiography revealed diffused calcified atherosclerotic changes in the supraclinoid internal carotid and basilar arteries. Causes of intracerebral hemorrhage (ICH) are mostly attributed to vascular anomalies or hypertension [1]. However, in this case, there was no radiological evidence of acute major vascular occlusion, aneurysm, or vascular malformation. The patient survived COVID-19 pneumonia during the same admission without complications. And, was discharged home with an improved GCS of 13/15 off sedation, equal reactive pupils, and left-sided monoplegia. Subsequent CT follow-ups of the brain showed marked resolution of the hematoma. Given the radiological appearance of blood-casted cisterns on the initial CT, we cautiously followed the patient with GCS examinations and serial CT imaging of the brain, as subtle changes in the size of the cerebrospinal fluid spaces are the early signs of hydrocephalus, which in this case has never developed. In this case, the primary neurosurgeon opted for conservative management, as there was no indication for hematoma evacuation or external ventriculostomy drain. In this case, the blood-CSF cocktail molded the ventricular system, drawing its detailed anatomy. I believe this brain CT image is a "teachable coincidence" yet anatomically reproducible in this common condition. It also resembles a crested bird’s head most strikingly a Crax rubra (Figures 2, 3). This case report suggests the name “Crax rubra sign” for this finding.

## Conclusions

Intraparenchymal hemorrhage with intraventricular extension is a morbid diagnosis. A cast fourth ventricle is a well-known entity that carries poor clinical outcomes. This illustrative case report presents a serendipitous radiographic finding of internal brain anatomy made discernible by an unfortunate occurrence of ICH and IVH. Being reproducible, we also believe, serves as great bird biomimicry for the young radiologists to memorize. Obstructive hydrocephalus obviously does not develop in each case scenario. Therefore, this case sheds light on the idiosyncrasies of hematoma resolution in different patients and the CSF-blood chemical interaction in IVH cases. Research employing brain models is needed to study the blood-ependymal interface of the cast itself. To further understand the predictors of outcome and plan effective management.
